# Supplementary material for: Are carbon emissions trading and green financial instruments synergistic? -Comprehensive quantitative research based on content analysis
Source: PLoS One. 2024 Mar 7;19(3):e0298601. doi: 10.1371/journal.pone.0298601 (PMC10919601; doi:10.1371/journal.pone.0298601)
Supplement: S1 Data — (ZIP) [file pone.0298601.s001.zip › Data/地级市Z_F工作报告.zip!/║╙▒▒/▓╫╓▌╩╨/└┤╫╘╣1⁄2╓┌║┼í╛┬φ┐╦╩2╛▌═°í┐_2017.docx]

各位代表：

现在，我代表沧州市人民政府，向大会作工作报告，请予审议，并请各位政协委员和列席会议的同志提出意见。

一、本届政府工作回顾

2013年以来，面对复杂多变的宏观经济环境和艰巨繁重的改革发展任务，全市上下在省委、省政府和市委的坚强领导下，攻坚克难，团结奋进，圆满完成了十三届人大确定的各项目标任务，在全面建成小康社会进程中迈出坚实步伐。

综合经济实力快速提升。围绕稳中求进工作主基调，着力壮大经济总量，提高发展质量。全市地区生产总值由2012年的2811.9亿元增加到2016年的3533.4亿元、年均增长8.1%，经济总量从全省第四跃居第三；一般公共预算收入由142.6亿元增加到220.4亿元、年均增长11.5%。规模以上工业增加值达到1308.2亿元、年均增长9.2%，固定资产投资3481.1亿元、年均增长16.8%，社会消费品零售总额1227.8亿元、年均增长12%。经济发展更趋稳健、活力日益增强，位列中国经济实力百强城市第57位。

沿海开发实现重大突破。围绕进一步扩大渤海新区影响力、带动力，大力推进港口国际化、产业集群化。20万吨级航道、20万吨级矿石码头、邯黄铁路建成投用。黄骅港口岸扩大开放获国务院批复，综合保税区一期工程基本完工，开通“黄—新—欧”国际物流班列和直航东南亚国际航线，启动与天津港全面合作。2016年完成港口吞吐量2.45亿吨，集装箱60.15万标箱，分别是2012年的1.88倍和6倍，增速居全国沿海主要港口首位，成为我国第一大能源港。中国一重冷轧薄板、中海油中捷石化油品质量升级、北汽60万辆微车等一批重点项目竣工投产，新一轮沿海开发建设热潮正在兴起。

中心城区功能日益完善。围绕提升品位、增强辐射带动能力，推进实施了一批重点建设项目。“一场五馆”、高铁西站投入使用；黄河路快车道、维明路立交桥建成通车；建成区绿化覆盖率达到36.9%，获国家园林城市称号和省人居环境进步奖。新建、改造城区道路67公里，供水、供热、燃气管网255公里；新增精品小区49个、商品住宅面积624万平方米；建成区扩大了33平方公里。新华物流产业园、中国国际机器人产业园加快建设，沧州开发区汽车产业园、运河区激光产业园从无到有、迅速壮大，高新技术产业和高端服务业支撑能力进一步增强。中心城区实现地区生产总值占全市比重超过20%。

县域发展活力明显增强。围绕城乡一体、协调发展，做大县城规模，做强县域经济，做美城乡环境。全市销售收入超百亿元产业集群达到9个。河间“国家再制造产业示范基地”获批建设，黄骅、南皮列入国家级电子商务进农村综合示范县。全部财政收入超十亿元县（市）达到11个，任丘超过百亿元。县城建设步伐明显加快，13个县（市）成功创建省级园林城市（县城），肃宁获国家园林县城称号。美丽乡村建设扎实推进，完成1506个省级重点村改造提升任务。全市城镇化率达到50.6%。

结构调整取得实质进展。围绕调结构、促转型，突出创新驱动，加快供给侧结构性改革。累计实施亿元以上技改项目885个，完成投资2053亿元。汽车制造、生物医药、激光研发与应用等新兴产业异军突起，成为经济发展的新亮点。机械制造业完成增加值占规上工业比重超过石化产业，成为第一支柱产业。科技型中小企业、科技小巨人企业分别达到8220家和300家，均居全省前列。新增规模以上服务业企业285家，达到444家，服务业对经济增长贡献率超过第二产业。现代农业稳步发展，省级现代农业园区达到10家，渤海粮仓农业科技示范园晋升为国家级园区，全市农业产业化率达到64.5%。三次产业结构由2012年的11.3：52.6：36.1调整为8.7：49.5：41.8。

改革开放空间不断拓展。围绕进一步释放和增强发展活力，持续深化改革、扩大开放。取消全部行政监管和非行政许可审批事项，下放市本级行政权力事项330项，肃宁“四级网上审批与便民服务”典型经验在全国推广。全面建立“四个清单”“三级平台”“两个代办”制度，政务服务中心、公共资源交易中心规范运转。在全省率先推行“六个零”企业登记和“三证合一、一照一码”制度，新增市场主体19.3万户，年均增长24.8%。引进华夏、燕赵等12家银行和14家保险机构，全市存贷比达到57.6%，比2012年提高10.5个百分点。新增上市企业103家，总数达到135家。成功举办中国沧州（捷克）投资环境推介会、海外侨商沧州行等系列大型招商活动。累计利用外资17.9亿美元，完成外贸进出口112.7亿美元，年均增长12.2%和5.1%。

协同发展迈上新台阶。围绕抢抓国家战略机遇，积极作为、多点出击，全方位对接京津、承接转移。全市京津合作产业项目超千个，总投资3355亿元。去年10月18日，北京现代沧州工厂正式量产，成为区域协同发展的典范。北京生物医药产业园71家企业签约入驻，22家开工建设，开创异地监管新模式。与天津滨海新区签订战略合作协议，津冀（沧州）产业转移升级示范区顺利启动。高新区明珠商贸城成为北京“动批”“大红门”“八里桥”批发市场外迁承接地。我市3所三甲医院分别与北京301、天津肿瘤、北大肿瘤医院实现牵手合作，北交大海滨学院、北京中医药大学东方学院、北航教育园建成招生。协同发展“沧州剧场”风生水起、渐入佳境。

生态环境质量持续改善。围绕生态优先、绿色发展，精准发力、重拳出击，全力打好大气污染治理攻坚战。压煤、降尘、控车、减排、增绿等专项行动深入实施，购置新能源公交车1693辆，淘汰黄标车15.8万辆，取缔全部366座实心粘土砖瓦窑，淘汰改造燃煤锅炉2126台，压减燃煤109.7万吨。积极推动气（电）化工程，散煤燃烧治理替代工作持续开展。2016年，全市空气质量达标天数207天，PM2.5、PM10分别比2013年下降32.4%和14.8%。集中式饮用水源地水质达标率稳定保持100%，形成地下水压采能力4.38亿立方米。全市累计新增造林面积161万亩，森林覆盖率达到29.5%。

民生保障水平不断提高。围绕为民谋利、解民所忧，不断增加财政投入，办成了一批关系群众切身利益的好事、实事。城乡居民可支配收入分别达到28605元和11340元，比2012年增长37.5%和50.9%，城镇累计新增就业30.95万人。全面打响脱贫攻坚战，积极推进贫困县摘帽出列工作，447个贫困村实现脱贫退出。完成机关事业单位人员基本工资标准调整，城镇和农村低保标准分别比2012年提高79%和105%，城乡居民社会养老保险合并实施走在全省前列，农村互助幸福院、城市社区养老服务站覆盖率分别达到72%和83%。开工建设保障性安居工程8.1万套，城镇低收入住房困难家庭实现应保尽保。南水北调中线2条干渠和8条水厂以上输水管道如期完工，惠及人口400万。

社会事业取得全面进步。围绕补齐短板、均衡协调，大力推进社会事业发展。新建标准化中小学382所，河北工专成功升本，新一中建成投用。市直5家公立医院全部取消药品加成，国家分级诊疗试点工作稳步实施。成功举办第十四届省运会、第九届中国·沧州国际武术节，以及第十四、十五届中国吴桥国际杂技艺术节沧州主会场。国家公共文化服务体系示范区创建工作扎实推进，实现全国双拥模范城“四连冠”。安全生产形势总体平稳，社会治安形势持续向好，公众安全感和群众满意度逐年上升。民族宗教、外事侨务、人民防空、气象地震、史志档案、妇女儿童、老龄、残疾人等各项事业都取得了新成绩。

各位代表，2016年是本届政府的收官之年。一年来，面对不断加大的经济下行压力，我们坚持立足优势、趋利避害、积极作为，巩固了平稳较快发展的良好态势；面对发展中的困难挑战，我们坚持照单全收理旧账，突出问题导向，强化责任担当，有力有效地化解了矛盾和风险；面对经济社会发展中的差距短板，我们坚持“齐头并进不落项、主次轻重要分明”，把握平衡点、狠抓关键点，圆满完成了各项既定任务，主要经济指标增速保持全省前列，实现了“十三五”良好开局。全市地区生产总值增长7.9%，全省第二；规模以上工业增加值增长7%，全省第一；固定资产投资增长12.2%，全省第三；全部财政收入增长7.4%，全省第五；社会消费品零售总额增长10.7%，全省第三。在推进经济社会发展的同时，我们更加重视加强政府自身建设，深入贯彻落实十八届六中全会精神，扎实开展“两学一做”学习教育和机关作风整顿活动，施政能力和服务水平得到不断提升。全年办理人大代表建议151件、政协提案368件，按时办复率100%。

各位代表！本届政府取得的成绩是省委、省政府和市委正确领导的结果，归功于760万沧州儿女的团结拼搏，得益于历届政府打下的坚实基础，离不开市人大、市政协的监督支持。在此，我代表沧州市人民政府，向全市人民，向人大代表、政协委员，向各民主党派、工商联、人民团体和各界人士，向驻沧解放军、武警官兵和政法干警，向所有关心支持沧州发展的同志们、朋友们，致以崇高的敬意和衷心的感谢！

在总结成绩的同时，我们也清醒地看到，沧州发展仍处于爬坡过坎阶段，工作中还面临不少困难和问题。主要是：综合经济实力大而不强，经济发展质量和效益亟待提升；产业结构不尽合理，转型升级任务仍然较重；重点领域和关键环节改革的力度不大，对外开放的程度不高，发展环境仍需优化；城市规模不大，辐射带动能力不强，城镇化率较低；民生和社会事业存在短板，人民群众生活水平和公共服务水平还不高；大气污染防治形势严峻，生态建设仍需加强；一些政府部门和工作人员担当意识、服务意识不强，存在工作落实不到位、政策执行不严格和懒政怠政等问题。对此，我们一定高度重视、认真研究解决。

二、今后五年的奋斗目标和主要任务

未来五年是沧州乘势而上、跨越赶超的重要战略机遇期，省、市第九次党代会胜利召开，描绘了未来五年发展的宏伟蓝图，鼓舞人心、催人奋进。新一届政府将以“撸起袖子加油干”的饱满精神，激情工作，勇往直前，以更大的决心和力度，创造沧州速度、浓厚沧州气场、提升沧州特色、叫响沧州品牌，努力在走新路、求跨越、奔小康的征程中展现新作为。

今后五年政府工作的总体要求是：在以习近平同志为核心的党中央坚强领导下，全面贯彻党的十八大和十八届三中、四中、五中、六中全会精神，以邓小平理论、“三个代表”重要思想、科学发展观为指导，深入学习贯彻习近平总书记系列重要讲话精神和治国理政新理念新思想新战略，统筹推进“五位一体”总体布局，协调推进“四个全面”战略布局，认真贯彻创新、协调、绿色、开放、共享发展理念，积极适应和引领经济发展新常态，大力推进供给侧结构性改革，坚定走加快转型、绿色发展、跨越提升新路，解放思想、改革创新、奋发进取、乘势而上，为全面建成小康社会，加快建设创新驱动经济强市、生态宜居美丽沧州而奋斗。

主要工作目标是：在确保完成市第九次党代会提出的综合经济实力、创新发展能力、城乡环境质量、社会文明程度、人民生活水平“五个全面提升”的基础上，力争全市地区生产总值、全部财政收入和一般公共预算收入，在全省排位实现“追二、争三、夺四”的目标，分别达到5500亿元、800亿元和400亿元以上。重点推进六大战略任务。

（一）聚力创新、加快转型，让沧州发展更具竞争实力。建设创新驱动经济强市，必须紧紧抓住创新“第一动力”、把握转型升级“主攻方向”，推进发展动能“由旧向新”转变。围绕创建国家创新型城市，进一步加大政策支持力度，全力构建创新生态系统，不断提升研发经费支出占生产总值比重、高新技术产业占规模以上工业比重，以及科技进步对经济增长的贡献率。大力改造提升石油化工、管道装备、机械制造、食品加工、纺织服装“五大”传统产业，加快向精细化、高端化、智能化、循环化方向迈进。着力推进高端装备制造、新能源和清洁能源、生命健康、电子信息、通用航空、节能环保、再制造“七大”新兴产业，促其裂变增长、挑起大梁。加快发展现代服务业和现代农业，拓展新领域，催生新业态，推动现代服务业向专业化延伸、高品质提升，现代农业“接二连三”发展，实现产业结构的战略性转变。

（二）推进协同、开放合作，让沧州发展更具生机活力。3月28日，中共中央、国务院印发通知，决定设立雄安新区，这是以习近平同志为核心的党中央深入推进京津冀协同发展的一项重大决策部署，是千年大计、国家大事，必将对河北乃至全国贯彻落实新理念推动新发展，产生重大而深远的影响。雄安新区覆盖任丘白洋淀区域，与沧州近在咫尺，今后一个时期将是我们借势雄安新区开发建设、抢抓京津冀协同发展机遇最为宝贵和紧要的时期。一方面，我们要按照中央和省委的有关要求，全力支持好服务好新区建设；另一方面，要充分利用中央在河北设立雄安新区的机遇，以更高的标准和水平，搭平台、促合作、上项目、育产业，加快发展好自己，努力让沧州在协同发展大幕下放射出愈发夺目的光彩。坚持把全方位对接京津与实现自主发展结合起来，在深度承接产业转移与功能疏解的同时，围绕京津需求，放大自身优势，做好打造京津特殊需求功能区文章，为沧州的发展找到新的支撑点、赢得更大主动权。把发挥紧靠天津优势与推进同城发展结合起来，加快津冀（沧州）产业转移升级示范区建设，推动渤海新区纳入天津自贸区延伸区，探索与天津形成协作配套、互动发展新模式，最大限度发挥一体化、同城化效应。把瞄准京津与放眼全球结合起来，坚持对内与对外开放并举，引进来和走出去并重，积极融入“一带一路”战略，把沧州开放的大门敞得更开，让沧州与世界的互动更加精彩。

（三）保护生态、优化环境，让沧州发展更具特色魅力。生态环境污染是区域发展不可承受之痛，我们必须坚守生态和发展两条底线，像保护眼睛一样保护生态环境，科学治污、精准治污、依法治污、铁腕治污。坚持“标本兼治、冬病夏治”，坚决打赢蓝天保卫战，让蓝天一年比一年多起来。以创建国家生态园林城市为抓手，下决心做好增绿文章，着力构建“一海、九河、百园、千路、万座湖”的大生态格局，全市森林覆盖率达到33%以上，让绿色成为沧州最厚重的底色。“子规夜半犹啼血，不信东风唤不回”。我们一定义无反顾、勇往直前、奋力攻坚，努力建设水天人文共生、生态绿色宜居的美丽沧州，向人民群众交出合格答卷。

（四）简政放权、深化改革，让沧州发展更具内生动力。简政放权是政府自身的一场深刻革命。在生产要素加速流动、各地竞相发展的形势下，沧州尤其需要拿出比以往任何时候都要大的决心和力度，推动改革、夯基垒台、扎好“营盘”、释放红利。在统筹推进财税、金融、科技、国资国企等重点领域和关键环节改革的同时，以全省着力优化营商环境为契机，大力推进“放管服”改革，进一步清除阻碍发展的“减速带”，打通服务群众的“中梗阻”。学习借鉴先进地区“最多跑一次”的改革经验，探索推进行政审批“一次到位”、便民服务“零上门”机制，减权、放权、治权，便民、利民、惠民，用政府权力的“减法”换取市场活力的“加法”，全力打造一流营商环境，真正让沧州成为企业向往、产业聚集的“金色大厅”。

（五）完善路网、做美城镇，让沧州发展更具综合承载力。基础设施是城市建设的“底盘”，具有带动经济社会发展的“乘数效应”。我们要牢牢抓住国家加快基础设施建设的“黄金窗口期”，全力构建以公路、铁路、海运、航空为骨架的大交通体系，形成连接京津、贯通周边的高速公路网、高速铁路网，推动县县通高速向县县通铁路迈进。重点实施好环渤海、保沧、霸商、津承沧城际铁路和邯港、曲港、京沪北延、任德高速公路等项目，努力将沧州打造成为“轨道上京津冀”的重要枢纽，实现“高铁后时代”的跨越腾飞。畅通与省会的交通联系，缩短两地时空距离，是全市人民的共同期盼。我们将举全力推进石衡沧港城际铁路建设工程，力争早日建成通车，使之成为联通我省东西的交通大动脉、经济大动脉。按照2030版城市总体规划，加快行政区划调整，推进大沧州都市区建设，着力构筑“一核多星、一轴三带”的城镇化发展格局，全市城镇化率达到60%以上。统筹生产、生活、生态布局，精心规划每一寸土地，精细建设每一个片区，练好“内功”，提升“颜值”，在京津冀城市群中展示形象、彰显风采。

（六）保障民生、促进共享，让沧州发展更具向心凝聚力。“大道之行，天下为公”。站在新的发展起点，我们深知，全市人民求新求变、求富求强，对新一届政府满怀憧憬和希望。我们将坚持以人民为中心的发展思想，把增进人民福祉、促进人的全面发展作为一切工作的出发点和落脚点，实施新一轮民生幸福工程和脱贫致富奔小康工程，着力解决群众最关心最直接最现实的利益问题，在充实居民“口袋”、丰富公共服务、维护公共安全、提升文明程度上补短板，确保城乡居民人均可支配收入年均增长10%以上。巩固武术之乡、杂技之乡、好人之城、工笔画之城等品牌，守牢社会保障、社会救助、公平正义底线，努力把服务人民的实际成效书写在沧州大地上、印在群众的心坎上。

薪火相传，生生不息，新元肇启，继往开来。我们坚信，通过全市人民的共同努力，全面建成小康社会的目标一定能够早日实现，沧州崛起腾飞的梦想一定能够成为现实！

三、2017年主要工作

千里之行，始于足下。今年是实施“十三五”规划的重要一年，是供给侧结构性改革的深化之年，也是新一届政府贯彻落实省、市第九次党代会精神的开局之年。我们一定要按照建设创新驱动经济强市、生态宜居美丽沧州的目标定位，牢牢把握稳中求进工作主基调，奋力改革创新，提升质量效益，确保经济社会平稳健康发展。

经济社会发展的主要目标是：生产总值增长8.5%，规模以上工业增加值增长8%，一般公共预算收入增长9%，固定资产投资增长15%，实际利用外资增长10%以上，社会消费品零售总额增长10%，城镇、农村居民人均可支配收入分别增长8.5%和9%。主要污染物减排总量、环境质量控制等全面完成省考核目标。重点做好八个方面工作：

（一）着力保持经济平稳较快增长。坚持发展第一要务，进一步扩大需求、优化供给，夯实稳增长基础。

持续有效扩大投资。狠抓项目建设不动摇，深入实施领导分包责任制，引导优质资源向重点项目、重点产业集中，土地、资金等要素向能开工、能竣工的项目倾斜。积极扩大产业项目投资，鼓励支持社会领域投资，确保传化物流智能公路港、明珠服饰文化产业创意园等200个亿元以上项目开工建设、顺利推进，河北盛腾50万吨芳烃等100个亿元以上项目竣工投产，全年完成投资2000亿元以上。突出抓好总投资超5000亿元的30个重大战略支撑项目，加快北京生物医药产业园、核燃料产业园建设，力促海兴核电取得突破性进展。大力推广政府和社会资本合作（PPP）模式，进一步激发民间资本投资活力。

挖掘消费和外贸潜力。顺应消费需求变化趋势，大力发展文化旅游、健康养老、教育培训等幸福产业和服务经济，打造新的消费热点。进一步优化消费供给，培育1个国家级和4个省级知名品牌示范区，新增驰名商标5个、著名商标40枚、省名牌产品30个以上。实施“互联网+流通”行动计划，支持零售企业实体店数字化改造，开展电子商务进农村进社区工程，满足个性化消费需求。落实和完善鼓励出口政策，抓好省级外贸转型示范基地和进出口专业服务平台建设，加快发展服务贸易和服务外包，促进外贸优进优出，力争新增进出口超千万美元企业6家以上。

倾力服务实体经济。关注央企、关爱民企、关心外企，努力为企业发展提供“五星级服务”，构建“亲、清”新型政商关系。提高政银企合作成效，有效增加制造业和小微企业贷款，建立企业应急转贷机制，防止资金“脱实向虚”。推动“个转企、小升规、规改股、股上市”，年内新增规模以上工业企业150家以上、挂牌上市企业20家以上。支持引导企业不断提高核心竞争力，发扬“工匠精神”、打造“单项冠军”、培育“百年老店”。

（二）着力加快产业转型升级步伐。坚持把调结构、转方式放在更加重要的位置，通过优化存量和扩大增量“两手抓”，推动新旧动能转换。

做优做强传统产业。实施“双百工程”，扶优扶强100家重点企业，推进100家技改企业转型升级。以智能制造为主攻方向，推动两化深度融合，新增省级以上数字化车间3个、两化融合企业10家。全面推进全国质量强市示范城市创建活动，深入实施“增品种、提品质、创品牌”专项行动，建成国家皮毛产品质检中心、河北省节能炉具及散热器质检中心。

做实做大新兴产业。实施七大新兴产业发展“助力工程”，在项目引进、要素保障等方面给予重点扶持，力争高新技术产业增加值增长13%以上。加快河间再制造产业示范基地、北京科创园渤海新区产业基地、北航科技园、河北工大科技园、中捷通航产业园等项目建设，推动沧州开发区和黄骅汽车产业园、华北（沧州）高端智能装备产业园向千亿级目标迈进。支持“军转民”“民参军”，打造一批军民融合产业聚集区。

做特做专现代服务业。大力发展总部经济、楼宇经济，打造城市核心商圈、大型商业综合体和特色商贸街区。抓好渤海新区、沧东、肃宁三个省级物流园区建设，加快打造中国北方重要的现代物流基地。发展会展会议经济，举办好沧州·跨国公司国际论坛、汽车配件博览会等专业展会。深化与华为、微软、阿里巴巴等信息产业巨头合作，培育发展大数据、物联网、云计算产业。推动全域旅游和产业融合发展，用足用好白洋淀、大运河、渤海湾、武术和杂技五大顶级资源，加快建设大运河人文体验带、环渤海休闲度假带和白洋淀生态旅游带，叫响沧州旅游品牌，着力打造京津冀文化生态旅游首选目的地。

做精做细现代农业。加强粮食生产核心区建设，深入实施“渤海粮仓”科技示范工程和高标准农田建设，确保全市粮食产量稳定在86亿斤以上。着眼打造京津农副产品生产供应基地，积极推进农业供给侧结构性改革，依托畜牧、蔬菜、果品三大优势产业，组织实施农业品牌创建“112”工程，培育10个区域公用品牌、10个行业领军企业品牌和200个农产品品牌。加快现代农业园区建设，促进一二三产融合发展，确保年内新增7个省级现代农业园区，产业化率达到66%以上。

（三）着力实施创新驱动发展战略。坚持以便利、宽松、包容为导向，提升创新浓度，厚植创新沃土，加快培育创新型增长点。

强化产业科技创新能力。建立健全科技服务研发链条，新建省级以上产业研究院、工程技术研究中心、院士工作站、科技孵化器等创新服务平台19家。加快技术交易市场建设，依托线上线下、网内网外的有机融合，建立“供给库、需求库、案例库”，实现科技资源的开放整合与高效利用。开展10项重大产业技术集中攻关，承接一批国家和省重大科技成果转化项目。

发挥企业创新主体作用。启动新一轮科技型中小企业成长计划，抓好苗圃、雏鹰、小巨人和上市四大工程，年内新增科技型中小企业1800家以上、科技小巨人企业80家。实施高新技术企业后备培育工程，定向帮扶进入后备培育库的企业，新增高新技术企业50家。加强知识产权保护，支持企业参与标准制定和发明创造，年内万人发明专利拥有量达到1.5件。

健全科技创新体制机制。认真落实关于支持科技创新的系列措施，进一步完善科技成果转移转化机制，实行以增加知识价值为导向的分配政策，赋予科研院所和高校更大的科研自主权。建立以财政投入为引导、企业投入为主体、金融市场为支撑的多元化科技创新投入体系。

深度激发创新创业活力。坚持创新创业结合、孵化转化结合，推广众创、众包、众扶、众筹商业模式，发展创业投资和天使投资，突出抓好北京路“双创一条街”，新增省级众创空间4个、市级众创空间20个。实行更具灵活性和吸引力的人才政策，积极引进海内外科技领军人才和创新团队，让沧州成为有志之士施展才华、成就梦想的“黄金舞台”。

（四）着力推动协同发展、沿海突破。坚持打好京津、沿海“两张牌”，把临北京、靠天津、沿渤海的优势进一步转化为发展优势、竞争优势。

加快对接京津、区域融合。围绕互动交流多元化、区域市场一体化、社会服务同城化、交通运输便利化，强化产业合作和园区共建。着力打造沧州现代产业基地、渤海新区新型工业化基地、任丘石化产业基地三大对接平台，加快推进京南国家科技成果转移转化示范区、北交大轨道交通综合研发和试验基地、津冀（沧州）产业转移升级示范区、京津现代服务业转移基地建设。抓住与天津滨海新区签订合作协议的契机，大力推进滨海新区相关产业向沧州转移，培育滨海新区南部产业承接和支撑基地。瞄准中关村和科研院所、高等院校，全方位对接京津创新资源，加快“京津孵化、沧州转化”。积极承接非首都功能疏解，进一步深化教育、医疗等领域的合作。全力推动保沧、津承沧等城际铁路前期工作，确保石衡沧港城际铁路9月份开工建设。

深化陆海统筹、港腹联动。围绕发挥渤海新区龙头带动作用，进一步完善港口功能，提升运营能力，加快建设现代化国际贸易综合大港，力争总投资218亿元的19个专业码头顺利开工，全年吞吐量达到3亿吨，集装箱100万标箱，确保国家批准设立黄骅港综合保税区。大力发展临港产业，构建以石油化工、冶金装备、港口物流等传统产业为基础，以新能源、新材料等新兴产业为支撑的临港产业体系。围绕放大国家级开发区政策效应，加快推进临港经济技术开发区整合，进一步完善“一区多园”管理模式。加强与腹地沿线城市、大企业大集团的战略合作，加快“内陆港”、物流基地建设，扩大以集装箱为重点的海铁联运、多式联运。大力开辟国际班列和国际航线，拓展加密国际贸易通道，增强“东出西联”辐射带动能力。抓好基础和配套设施建设，打造港城联动、产城融合的现代化滨海新城。

（五）着力推进新型城镇化建设。坚持以人的城镇化为核心，围绕融入京津冀世界级城市群，全面提升城市建设水平，促进城乡统筹、协调、一体化发展。

提升中心城市能级。着眼东西部均衡发展，支持新华区“东扩南进”、拉大框架，运河区完善功能、丰富内涵，不断增强人口集聚能力。围绕创建全国文明城市、生态园林城市，打造智慧城市、海绵城市，实施总投资422亿元的145个重点城建工程。新建续建南北绕城公路、永济路提升改造等46条城市道路，确保九河路全线贯通。启动天成名著、保利花园等13个小区规划路项目，改造小街小巷26.5万平方米。推进10.5公里地下综合管廊试点，对水月寺、清真寺等重点片区28条道路进行雨污分流改造。努力全面消除城市黑臭水体，打造生态水系景观。加快第二水源及配套管网、运西污水处理厂二期建设，启动公墓搬迁工作。新建高新区、姚庄子和东骏百川3个菜市场，青少年宫、妇女儿童活动中心竣工投用。

推动县城扩容提质。按照“小县大县城”、产城教融合发展思路，深入实施城乡规划提升、基础设施建设、环境容貌整治等八项攻坚行动，力争黄骅、东光建成国家园林城市（县城），实现全市省级文明县城全覆盖。依托历史文化、产业基础、自然资源优势，建成一批服务县城、带动农村的商贸强镇、旅游名镇、特色小镇。进一步延伸产业链条，壮大特色产业规模，促进县域经济增比进位。

优化城市综合管理。完善提升城市管理委员会组织功能，统筹协调城市规划、建设和发展，推进城市管理全覆盖、精细化、长效化。深入开展城市环境容貌综合整治行动，加大治违、治脏、治乱力度，全面提升城市整体环境质量。加快推进乡改镇、村改居和户籍制度改革，力争常住人口城镇化率提高2个百分点。

改善农村环境面貌。以美丽乡村建设12个专项行动为抓手，统筹规划、分类推进、连片打造，高标准完成590个省级重点村改造提升任务，促进产业、文化、旅游“三位一体”发展，打造“高颜值”特色乡村。加快城乡供气、供水、垃圾处理、污水处理、公共交通一体化进程，促进城乡统筹发展。

（六）着力深化改革扩大开放。坚持改革不停顿、开放不止步，以更大的力度推动各项重点措施落地，进一步增强发展动力和活力。

深入推进供给侧结构性改革。针对新情况新问题，完善政策措施，推进“三去一降一补”取得更大进展。运用市场、标准、法治等手段，严控过剩行业新上产能；加大小产业群治理力度，推动企业向园区集中，积极引导企业通过兼并重组、靠大联强、改造提升，实现脱胎换骨式的转变。坚持住房的居住属性，加强市场调控和监管，持续增加住宅用地，严格规范开发、销售、中介等行为，让房价回到合理区间，确保房地产市场平稳健康发展。规范政府举债行为，支持企业去杠杆、债转股。落实国家减税降费的政策要求，进一步挖掘企业税费、生产要素、制度性交易等降成本空间，切实减轻企业负担。加大补短板力度，努力在公共服务、文明进步等方面实现新提升。

推动重点领域改革取得突破。深化国资国企改革，积极发展混合所有制经济，切实做好“三供一业”分离移交工作，推进经营性国有资产集中统一监管。深化财税体制改革，做好“营改增”后续工作和开征环境保护税前期工作。深化金融改革，组建金融控股公司，加快农村信用社改革发展；大力发展政府引导基金，年内基金总数达到28支以上。深化农业农村改革，完善农村土地“三权分置”办法，推进农村集体产权制度改革，赋予农民更多的财产权。全面完成市级机关内设机构改革，积极推进供销社综合改革。

拓展对内对外开放广度深度。充分利用国际国内两个市场、两种资源，瞄准世界500强、行业领军企业、大型财团和业内知名专家，加强交流对接，促进战略合作，争取洽谈引进100家大企业大项目。充分发挥招商中介、专业化招商团队、猎头公司作用，广泛开展宣传推介和海内外人才引揽工作，实现招商引资与招才引智的有机结合。

壮大提升开发区规模层次。深化机构编制、人事薪酬、行政审批和投融资四项改革，突出基础设施上层次、招商引资上项目、经济发展上水平三大任务，确保全部财政收入、固定资产投资等主要指标增速达到15%以上。推动渤海新区、沧州高新区创新引领、率先崛起，申报国家级新区和国家级高新区；力促沧州、任丘、河间、泊头开发区，打造品牌、跨越发展，建设转型升级示范区；支持盐山、青县等开发区，扩大规模、增比进位，建设特色产业聚集区、区域发展增长极。

打造一流营商环境。以更大力度简政放权，推进审批流程再造，全面开展“减证便民专项行动”和“四级网上审批”规范化标准化建设。进一步完善清单管理制度，加快形成“综合清单+专项责任清单”管理体系。市、县两级全面建立行政审批局，打造“一站式审批、一条龙服务”新平台。推进企业登记五证合一、证照联办，规范中介服务和收费。认真执行“双随机、一公开”监管机制，严肃查处不作为、乱作为、吃拿卡要等行为。

（七）着力改善生态环境质量。坚持绿色、低碳、循环发展，深入实施蓝天行动、碧水行动、净土行动，努力在治理污染、修复生态中加快营造良好人居环境。

持续不断改善大气质量。坚持工程减排结构减排并举，城镇农村齐抓，白天黑夜严管，坚决控制污染物排放，确保PM2.5平均浓度下降10%左右。建立污染源排放清单和违法排污企业“黑名单”制度，严控挥发性有机物污染。加快普及城市集中供热，深入实施气（电）化工程，抓好农村散煤替代和清洁能源高效集中利用。强化机动车尾气治理和燃油品质管理，大力整治建筑施工、道路、渣土运输等扬尘污染。全面禁止露天焚烧，实现全市农田秸秆综合利用全覆盖。完善重污染天气应急预案，及时预报预警，科学精准应对。强化环保督察监察和环境质量考核，落实党政同责、一岗双责，保持上下联动、齐抓共管、网格监控、追责问责的高压态势。

做好水土污染防治工作。严格落实国家“水10条”、省“水50条”和“河长制”，加快工业园区和乡镇污水处理厂建设，加强子牙新河、南运河等重污染河流的治理，以及大浪淀水库等饮用水源地的保护。严格控制工业污染物排放和农村面源污染，促进土壤资源永续利用。

全面加强生态修复保护。统筹实施东部盐碱地绿化和南部、西部沙化土地治理工作，年内每个县（市）至少建成2个万亩绿化大方或生态林场；全市新增造林面积40万亩，森林覆盖率达到30%以上。实施河流水系、近岸水域生态修复工程，加强南大港、海兴等国家和省重点湿地的保护与修复。持续抓好地下水超采综合治理，确保完成省下达的任务目标。

（八）着力增进民生福祉。坚持从事关群众切身利益的突出问题入手，增投入、惠民生、促共享，让人民群众有更多获得感、幸福感。

大力推进脱贫攻坚。突出精准扶贫、精准脱贫主线，扎实推进“五个一批”工程，因地制宜发展特色扶贫产业，年内7.9万贫困人口稳定脱贫、227个贫困村脱贫退出。加大涉农资金整合力度，推广“政银企户保”金融扶贫模式。做好对口帮扶、驻村帮扶和社会帮扶工作，严格脱贫退出机制，坚决防止数字脱贫、虚假脱贫。

加大社会保障力度。实施更加积极的就业扶持政策，统筹解决好高校毕业生、城镇困难人员、退役军人等重点人群就业问题，全年城镇新增就业6万人。推进社保扩面提标，新增企业职工养老保险参保人数1.5万人。强化医疗、医保、医药“三医联动”，深化公立医院改革，推进分级诊疗制度，加快医疗联合体建设，打造健康沧州。落实城乡低保与物价上涨联动机制，确保困难群众基本生活。

协调发展社会事业。坚持教育优先、均衡发展，扩大学前教育规模，逐步消除义务教育学校大班额现象，加快普通高中优质特色发展，提升职业教育服务产业发展能力和高等教育发展水平。精心组织好沧州建州1500周年、第十六届中国吴桥国际杂技艺术节等系列文体活动，完成第三批国家公共文化服务体系示范区创建任务。深入实施全面两孩政策，改革完善计生服务管理。

提高社会治理水平。强化公共安全意识，健全安全生产责任制和隐患排查治理体系，深入开展危险化学品、油气管网、道路交通、建筑施工等高危行业和领域专项整治，落实食品药品安全监管“四个最严”，有效防范公共安全事件和事故。加强和创新社会治理，做好各类风险化解和信访维稳工作，用心用情用力解决群众利益诉求，打造平安沧州“升级版”。加强国防教育和后备力量建设，巩固军政军民团结的好局面。充分发挥工会、共青团、妇联等人民团体桥梁纽带作用。继续做好民族宗教、外事侨务、援疆援藏、红十字会、老龄、残疾人等工作。

民之所望，施政所向。今年我们将重点办好10件民生实事：一是加大教育投入力度，完成师院附中新建项目主体工程，确保十三中扩建一期年内竣工，新改扩建中小学382所、幼儿园72所，完成沧州市职业教育公共实训基地建设。二是建设保障性安居工程，全市开工各类棚户区改造住房11373套，基本建成棚改和公租房1.5万套。三是大力发展养老事业，全市新建养老机构8家、社区养老服务站16个、农村互助幸福院260个，新增养老床位3200张。四是改善乡村通行条件，新建、改造农村公路174条。五是实施基层中医药服务“百镇千村千人”计划，新建国医堂44个，全市覆盖率达到80%。六是加快南水北调水源切换工作，6月底前确保受水区居民喝上长江水。七是建设全民健身惠民工程150个、美丽乡村农民体育工程400个、中心城区体育健身示范工程20个，新增健身场地15万平方米。八是推动老城区集中供热扩容，实施中心城区东西部热网连通改造项目，新增供热面积240万平方米。九是打造优美城市环境，市区新建改造动物园、运河景观带一期和植物园二期等7个园林绿化项目。十是启动智慧泊车项目，新建、整合、规范中心城区停车场32处，新增泊位2400个。

四、全面提升政府建设水平

围绕“为民、务实、清廉”和“忠诚、干净、担当”要求，不断加强和改进政府工作，努力建设人民满意的服务型政府。

讲规矩、守底线，切实做到从严治政。牢固树立政治意识、大局意识、核心意识、看齐意识，严格执行《准则》和《条例》，认真落实全面从严治党主体责任，切实加强党风廉政建设和反腐败斗争。严格遵守中央“八项规定”，强化行政监察和审计监督，驰而不息整治“四风”。加大行政问责追责力度，坚决查处领导干部履职不尽责、不到位和失职、渎职等行为。

讲法治、强规范，切实做到依法行政。把政府工作全面纳入法治轨道，依法履行政府职能，不断提高政府工作人员法治思维和依法行政能力。严格落实重大行政决策法定程序，推动政府科学决策、民主决策。主动接受人大法律监督、政协民主监督、社会和舆论监督，广泛听取各民主党派和社会各界的意见建议。全面推进政务公开、信息公开，增强决策透明度，让行政权力在阳光下运行。

讲担当、重落实，切实做到实干实政。大力倡导有心尽责、有能尽职，真抓实干、埋头苦干，以创新精神破解制约发展的矛盾和问题，努力干出新业绩、开创新局面。改进文风会风，简化办事程序，提高行政效率，集中精力谋大事、抓实事。建立健全正向激励机制和容错纠错机制，让政府工作人员想干事、敢干事。坚持言必信、行必果，认准的事情一抓到底、承诺的事项坚决兑现，切实做到果敢担当、雷厉风行。

各位代表！发展没有坦途，奋斗成就精彩。今年是沧州建州1500周年，也是沧州解放70周年，勤劳智慧的沧州人民创造过光辉灿烂的历史，也一定能够在加快转型、绿色发展、跨越提升新路上谱写新的辉煌。让我们更加紧密地团结在以习近平同志为核心的党中央周围，在中共沧州市委的坚强领导下，解放思想，改革创新，奋发进取，乘势而上，为加快建设创新驱动经济强市、生态宜居美丽沧州而努力奋斗，以优异成绩迎接党的十九大胜利召开！
